# Identification of cognitive load-dependent activation patterns using working memory task-based fMRI at various levels of difficulty

**DOI:** 10.1038/s41598-023-43837-w

**Published:** 2023-09-30

**Authors:** Seyedeh Naghmeh Miri Ashtiani, Mohammad Reza Daliri

**Affiliations:** 1 Biomedical Engineering Department, School of Electrical Engineering, Iran University of Science and Technology (IUST), Tehran, Iran; 2 School of Cognitive Sciences (SCS), Institute for Research in Fundamental Sciences (IPM), Tehran, Iran

**Keywords:** Cognitive neuroscience, Working memory

## Abstract

Working memory, which is regarded as the foundation of cognitive processes, is a system that stores, processes, and manipulates information in short intervals of time that are actually needed for daily functioning. This study aimed to assess the brain activity of healthy controls (HC) while performing the N-back task, which is one of the most popularly used tests for evaluating working memory along with functional magnetic resonance imaging (fMRI). In this regard, we collected fMRI data from right-handed individuals in a 3.0 T scanner during the Persian version of the visual variant N-back task performance with three levels of complexity varied throughout the experiment (1, 2, and 3-back conditions) to increase the cognitive demands. The statistical parametric mapping (SPM12) software was used to analyze fMRI data for the identification of cognitive load-dependent activation patterns based on contrast images obtained from different levels of task difficulty. Our findings showed that as cognitive complexity increased, the number of significant activation clusters and cluster extent increased in several areas distributed in the cerebellum, frontoparietal lobes, insula, SMA, and lenticular nucleus, the majority of which are recognized for their role in working memory. Furthermore, deactivation patterns during 1-, 2-, and 3-back vs. 0-back contrasts revealed significant clusters in brain regions that are mostly described as being part of the default mode network (DMN). Based on previous research, our results supported the recognized involvement of the mentioned cortical and subcortical areas in various types or levels of N-back tasks. This study found that altering activation patterns by increasing task difficulty could aid in evaluating the various stages of cognitive dysfunction in many brain diseases such as multiple sclerosis (MS) and Alzheimer's disease by comparing controls in future studies to apply early appropriate treatment strategies.

## Introduction

The ability of the brain to function effectively in cognitive domains such as working memory, information processing efficiency, executive functioning, attention, and processing speed, which are the most commonly compromised functions, is known as cognitive functions. One of the most frequently reported cognitive challenges appears to be recalling recent events. Working memory, considered the foundation of cognitive processes, is a system that stores, processes, and manipulates information in the short bursts needed for daily function^1–4^.

The N-back task is a cognitive performance measurement task that is commonly used in neuroimaging studies to stimulate subjects' brain function. Kirchner was the first to introduce this task in 1958^5^. The N-back task involves functions such as attention control, decision-making, planning, speed of information processing, and so on. In the field of working memory, when performing this task, the most involvement is formed in the performance of the central executive system. Because this task involves both the maintenance and manipulation of cognitive information, it is well-suited to measuring working memory performance and has been widely used in this field in recent years. In the task's overall procedure, a series of stimuli (generally visual) is presented to the participants, and they must determine whether the currently presented stimulus is consistent with the N steps before it. This task is run with various numbers of N, and increasing N makes the task more difficult as cognitive load complexity increases. Thus, in the 1-back task (N = 1), the most recently presented stimulus is compared to the previous stimulus, whereas in the 3-back (N = 3), the most recently presented stimulus is compared to the previous three stimuli (in this paradigm, N can be 1, 2, or 3)^6,7^.

In functional magnetic resonance imaging (fMRI), the statistical relationship between different brain regions is determined using time fluctuations depending on the blood oxygen level. Studies using fMRI reveal patterns of activity in various brain regions while performing a particular task or while at rest, enabling researchers to compare brain function patterns elicited by different stimuli or populations of people^8–11^.

The popularity of the N-back task in functional neuroimaging studies is evidenced by the numerous studies in the field of working memory that have been published, including examining individual differences in measuring working memory performance in healthy subjects as well as special groups such as brain injury patients, substance abusers, people with brain disorders like depression, schizophrenia, multiple sclerosis (MS), etc.^12–22^. Owen et al.^20^ published the first meta-analyses of studies that used fMRI and the N-back task in adults. According to the results, the N-back task engages a set of brain areas, including the parietal and prefrontal cortical regions. In another meta-analysis of adults, Rottschy et al.^21^ used fMRI experiments on healthy subjects to investigate which brain regions are commonly active during working memory tasks. The main finding was an extensive bilateral fronto-parietal network that confirmed previous findings. Wang et al.^22^ also conducted a quantitative meta-analysis of 96 initial investigations of the N-back task based on different memory loads (1-back, 2-back) in healthy subjects. The fronto-parietal network is frequently activated throughout N-back studies, according to the activation likelihood estimation (ALE) method. In particular, the bilateral middle frontal gyrus, bilateral inferior parietal lobule, bilateral precuneus, left superior frontal gyrus, left anterior insula, and bilateral thalamus were all consistently activated across all the studies. Harvey et al.^14^ assessed changes in brain activity patterns of depressed patients and healthy subjects while performing the verbal version of the N-back task during fMRI scanning by varying working memory load to three levels (1, 2, and 3-back). While both groups showed activation of the prefrontal cortex, anterior cingulate, and parietal cortex according to results analyzed with SPM99 software^23^, depressed patients demonstrated greater activation within these regions than healthy subjects to keep the same level of performance. Furthermore, Rocca et al.^18^ used fMRI scans in a large cohort study at six European sites with data from MS patients and healthy controls (HC) while participants completed the N-back task under load conditions. In a comparison of the two groups, cognitively impaired (CI) MS patients had lower activations of several areas in the fronto-parieto-temporal lobes and lower deactivations of regions in the default mode network as task difficulty increased. In a recent study, Yaple and Arsalidou^3^ also used activation likelihood estimation to analyze fMRI data from children under 15 years of age during N-back tasks with two levels of difficulty. In their findings, consistency was found in frontoparietal areas known for their function in working memory and areas like the insula that aren't commonly emphasized as being part of the working memory network.

In this paper, we aimed to evaluate the brain activity patterns of healthy subjects related to cognitive function by manipulating task complexity. To detect these cerebral activation patterns, we designed a visually Persian version of the N-back task with three levels of cognitive demand. These identified patterns in healthy subjects can be used in future research in relation to the results of patients suffering from cognitive dysfunction across a range of neurological disorders to help us categorize cognitive impairments at various stages and analyze the effects of various treatments. Because cognitively abnormal patients appear to need more brain activation resources than controls to inhibit disorder manifestation and maintain comparable performance to HC when performing at lower cognitive loads, these activation resources may be constrained when performing at higher cognitive loads^14,24^.

The participants' characteristics, the task design, and procedures for collecting fMRI data will be described in the remaining sections of this paper. The steps for data analysis will then be described. Finally, the findings will be presented and discussed.

## Materials and methods

### Dataset description

Images of 12 healthy right-handed individuals (8 females and 4 males), with a mean age and standard deviation of 30.58 ± 4.72 years, were obtained using a 3.0 T Siemens Tim Trio MRI scanner at Imam Khomeini Hospital Complex, Tehran, Iran. Informed consent was obtained from all subjects taking part in the experiment.

There were two categories of images in this dataset: structural and functional. Structural images (3D T1-weighted and MPRAGE pulse sequence) were obtained with a matrix size of 256 × 256 along 176 sagittal slices with voxel dimensions of 1 × 1 × 1 mm^3^, flip angle of 7°, and time parameters of TE/TR = 3.44/1800 ms. Functional brain imaging was performed using an echo-planar imaging (EPI) sequence with a matrix size of 64 × 64 over 30 slices per volume and a slice thickness of 4 mm. These images had a resolution of 3 × 3 × 4 mm^3^ and were captured with a flip angle of 90, field of view (FOV) of 192 mm^2^, and TE/TR times of 30/2000 ms. The slice acquisition order was interleaved in an even–odd pattern for each individual's fMRI scanning, which contained 396 volumes.

Three of the 15 healthy subjects were excluded from the study for various reasons, including claustrophobia and failure to complete the scanning protocol properly.

### N-back task design

A parametric design was used to create the Persian version of the N-back working memory task, with different levels appearing in blocks at random. It should be noted that the N-back task in this study was performed with visual stimuli consisting of selected letters from the Persian alphabet.

**Figure 1:**
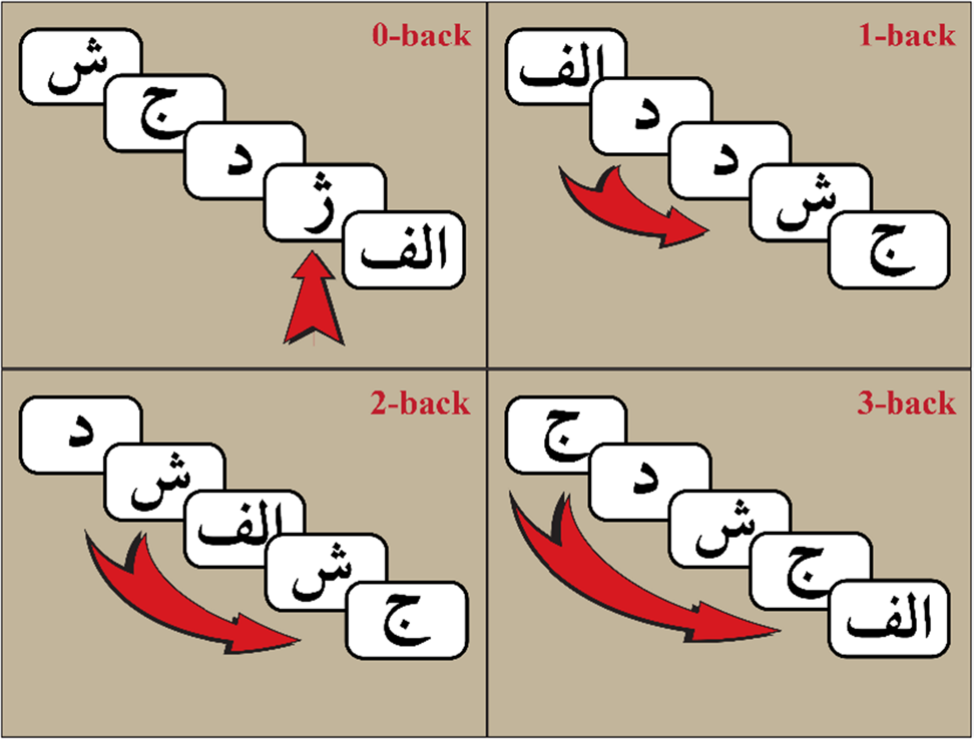
Visually designed Persian version of the N-back task with three levels of difficulty.

The task was to present the subject with a series of visual stimuli in a random order, and the subject had to determine whether the current stimulus was consistent with the N stimulus that came before it. Every 3 s, a new stimulus was displayed on a screen in the scanner room, which the patients could see through a mirror standard system on the scanner head coil. This task was performed in this study with various values of N ranging from 0 to 3. The larger the amount of N, the more difficult the task (cognitive load was increased). Thus, in a 1-back (N = 1) task, the last stimulus presented is compared to the previous stimulus; in a 2-back (N = 2) task, the stimulus presented is compared to two of its predecessors; and similarly, in a 3-back (N = 3) task, the last stimulus presented is compared to the previous three stimuli. The target letter in the 0-back (N = 0) task was always one specific letter (see Fig. 1).

Each block had 20 trials of 3 s each, for a total of 60 s, and was preceded by a 6-s instruction for each block. All blocks of 0-, 1-, 2-, and 3-back conditions were presented three times in random order, for a total stimulus length of 13 min and 12 s.

Participants completed the task training and practice phase prior to the fMRI scans, and after passing, entered the fMRI scanning phase under the N-back task performance. During the task execution, information such as the number of correct answers, incorrect answers, unanswered cases, and reaction time was yielded using the response box. Psychtoolbox-3 (www.psychtoolbox.org) was used to deliver stimuli, collect output from the response box, and save the results. Table 1 depicts the demographic characteristics and behavioral execution of participants.

**Table 1:** Demographics and N-back task performance of healthy controls. Values correspond to means and standard deviations.

	Sex	Age (years)	Education (years)	1-back ACC. (%)	1-back RT. (sec)	2-back ACC. (%)	2-back RT. (sec)	3-back ACC. (%)	3-back RT. (sec)
Healthy controls	8F/4M	30.58 ± 4.72	16.50 ± 3.60	98.58 ± 2.83	0.57 ± 0.14	93.54 ± 7.91	0.66 ± 0.14	77.58 ± 9.79	0.74 ± 0.23

*ACC* accuracy, *RT* reaction time, *F* female, *M* male.

### Data analysis

Data preprocessing and statistical analysis were performed using statistical parametric mapping (SPM12) software (https://www.fil.ion.ucl.ac.uk/spm). Registration with higher degrees of freedom and segmentation operation were used to analyze 3D T1-weighted scans.

The first step in preprocessing fMRI images before statistical analysis was to remove the first 3 volumes to ensure the steady state of the BOLD signal. Since different scans of the entire volume of the brain were not recorded at the same time, the next stage of preprocessing was the slice timing correction. Realignment of all images to the mean image to correct for subject motion, normalization into the Montreal Neurological Institute (MNI) space, spatial smoothing with a Gaussian kernel of 5 mm^3^, and temporal smoothing with a high-pass filter cutoff value of 264 were the next steps in preprocessing. There was no subject with head movement greater than half the voxel size (1.5 mm) to exclude from the subsequent statistical analysis.

After preprocessing, images were analyzed using a two-level random-effect approach under SPM12. In the first step, the time series of fMRI data regarding each participant were analyzed separately. The general linear model (GLM)^23^ was used to examine changes in BOLD contrast associated with the impacts of the N-back task performance on a voxel-by-voxel basis. Each subject's data was modeled using a first-level blocked task design convolved with a canonical hemodynamic response function (HRF), which also included motion parameters as regressors to evaluate specific effects by defining proper contrasts. For each subject, six linear contrasts were created: three 1-back, 2-back, and 3-back task conditions vs. 0-back; two 2-back and 3-back task conditions vs. 1-back; and a 3-back task condition vs. 2-back. Before proceeding to the second level, the activation/deactivation maps were validated in all subjects. Finally, regions displaying increased activation/deactivation with increasing task complexity (N-back load) were discovered using different levels from 0-back to 3-back. For individual contrast images, the statistical threshold was set at *p* < 0.05, with family-wise error (FWE) corrected for multiple comparisons at the cluster level.

**Figure 2:**
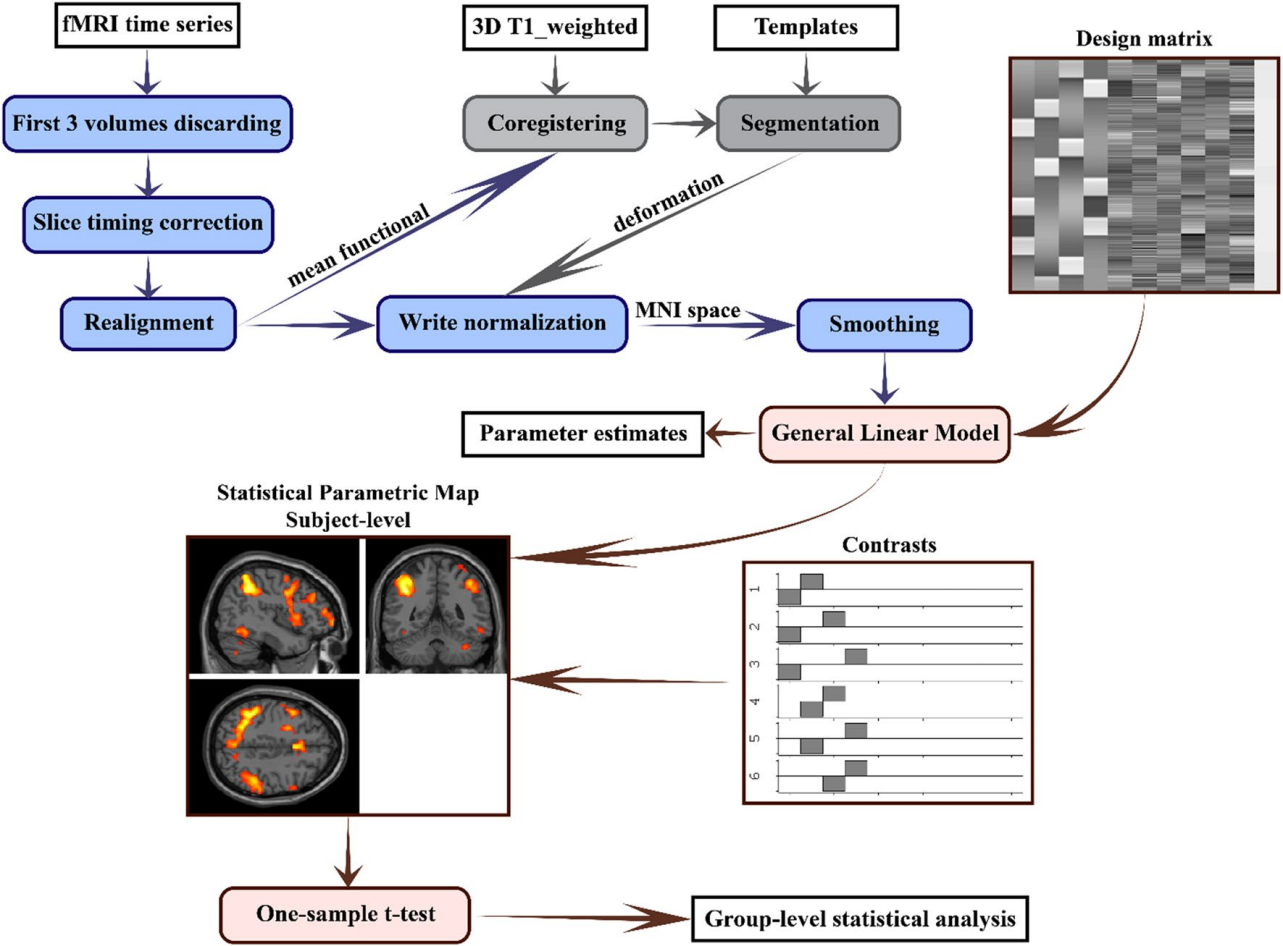
Scheme of the data analysis approach in the study. *3D*, three dimensional; *MNI*, Montreal Neurological Institute.

For the second-level statistics analysis, one-sample t-tests were used for the contrast images to identify significant clusters of activation/deactivation within the group during each complexity level of 1-back, 2-back, 3-back vs. 0-back, 2-back, 3-back vs. 1-back, and 3-back vs. 2-back. The results were displayed at *p* < 0.001, uncorrected, and as a whole-brain based on the third version of Automated Anatomical Labeling (AAL3) atlas (http://www.gin.cnrs.fr/en/tools/aal/) using the xjview tool (https://www.alivelearn.net/xjview). All of this study's proposed analysis steps are depicted in Fig. 2.

### Ethics declarations

All experimental procedures were carried out according to the institutional and/or national research committee's ethical standards, as well as the 1964 Helsinki declaration and its subsequent amendments or comparable ethical standards. The experimental protocols were also approved by Neuroscience & Neuroengineering Research Laboratory ethic committee of Iran University of Science & Technology (IUST).

## Results and discussion

**Figure 3:**
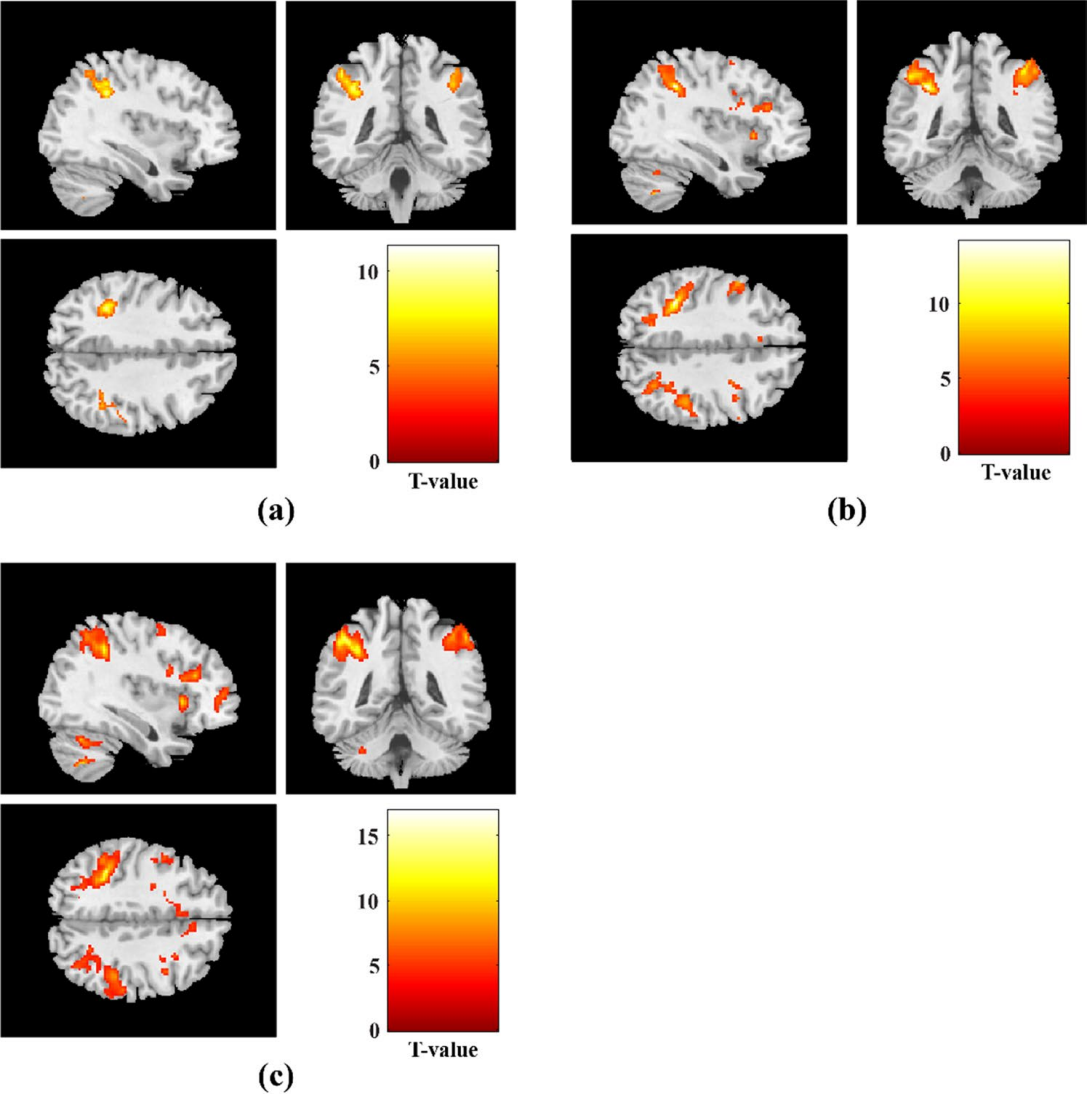
Significant clusters of activated brain regions during 1-back vs. 0-back, 2-back vs. 0-back, and 3-back vs. 0-back contrasts with increasing cognitive task load in healthy controls (one-sample t-tests, *p* < 0.001, uncorrected).

Figure 3 and Table 2 illustrate clusters of brain areas that were significantly activated during the N-back task while cognitive load increased from 1-back to 3-back vs. 0-back condition for within-group analysis. As can be seen, there were 7, 13, and 15 significant clusters of activation found respectively during the contrasts of 1-back, 2-back, and 3-back vs. 0-back with increasing task difficulty. The patterns of activation in all three mentioned contrasts were observed in the bilateral inferior parietal gyri (IPG), insula, right middle frontal gyrus (MFG), right superior frontal (dorsolateral) gyrus (SFG), some parts of the cerebellum, and supplementary motor area (SMA). In comparison to 1-back vs. 0-back, 2-back and 3-back vs. 0-back revealed a greater number of significant activation clusters with larger cluster extents. For contrasts of 1-back vs. 0-back, 2-back vs. 0-back, and 3-back vs. 0-back, respectively, 2 (1021 voxels total), 2 (2987 voxels total), 2 (3484 voxels total) parietal clusters, 1 (54 voxels total), 4 (1581 voxels total), 6 (3823 voxels total) frontal clusters, and 1 (35 voxels total), 3 (409 voxels total), 5 (895 voxels total) cerebellum clusters were significantly activated. With increasing cognitive task demand, the activation patterns during 2-back and 3-back vs. 0-back expanded to include additional brain regions such as cerebellum parts, triangular and opercular parts of inferior frontal gyri (IFG), left middle frontal, right superior parietal gyrus (SPG), right precentral gyrus, as well as right putamen and left pallidum.

**Table 2:** Clusters of brain regions that were significantly activated during 1-back vs. 0-back, 2-back vs. 0-back, and 3-back vs. 0-back contrasts with increasing task difficulty in a within group analysis (one-sample t-tests, *p* < 0.001, uncorrected).

Image contrast	Brain region	Side	Peak MNI coordinates (*x y z*)	Cluster extent	*T*-value
1-back vs. 0-back (activation clusters)	Inferior parietal gyrus (IPG)	L	− 48 − 42 54	546	11.29
	Supplementary motor area (SMA)	L	− 6 10 54	290	11.05
	Supplementary motor area (SMA)	R	4 18 50		8.50
	Insula (INS)	R	32 16 6	91	9.87
	Inferior parietal gyrus (IPG)	R	40 − 50 52	475	8.20
	SupraMarginal gyrus (SMG)	R	44 − 36 44		7.61
	Cerebellum_8 (CER8)	L	− 34 − 62 − 46	35	7.80
	Cerebellum_7b (CER7b)	L	− 24 − 68 − 44		5.99
	Precentral gyrus (PreCG)	R	34 0 48	101	7.71
	Superior frontal gyrus-dorsolateral (SFG)	R	28 10 54		6.25
	Middle frontal gyrus (MFG)	R	34 52 16	54	7.50
2-back vs. 0-back (activation clusters)	Inferior parietal gyrus (IPG)	L	− 38 − 44 40	1358	14.15
	Precuneus (PCUN)	L	− 10 − 68 54		9.79
	Inferior parietal gyrus (IPG)	R	40 − 42 52	1629	12.79
	Superior parietal gyrus (SPG)	R	16 − 60 58		10.85
	Middle frontal gyrus (MFG)	L	− 26 − 2 56	248	11.84
	Precentral gyrus (PreCG)	L	− 34 0 58		9.33
	Superior frontal gyrus-dorsolateral (SFG)	L	− 22 0 48		8.08
	Insula (INS)	L	− 28 22 4	145	11.00
	Inferior frontal gyrus-triangular part (IFGtriang)	L	− 40 28 24	687	9.52
	Precentral gyrus (PreCG)	L	− 54 8 36		8.74
	Cerebellum_6 (CER6)	L	− 26 − 66 − 26	175	9.43
	Middle frontal gyrus (MFG)	R	42 38 16	330	9.28
	Cerebellum_6 (CER6)	R	30 − 60 − 26	174	9.25
	Supplementary motor area (SMA)	R	6 16 50	567	9.09
	Supplementary motor area (SMA)	L	− 6 10 52		8.88
	Putamen (PUT)	R	30 18 2	172	8.78
	Insula (INS)	R	36 28 0		5.37
	Cerebellum_8 (CER8)	L	− 36 − 58 − 46	60	8.72
	Cerebellum_7b (CER7b)	L	− 32 − 68 − 48		6.20
	Middle frontal gyrus (MFG)	R	30 12 50	316	7.05
	Precentral gyrus (PreCG)	R	54 6 42	99	5.73
	Inferior frontal gyrus-opercular part (IFGoperc)	R	44 6 26		5.27
3-back vs. 0-back (activation clusters)	Cerebellum_6 (CER6)	L	− 28 − 64 − 30	360	16.84
	Inferior parietal gyrus (IPG)	R	48 − 36 50	1801	16.30
	Cerebellum_8 (CER8)	L	− 34 − 62 − 46	152	14.17
	Inferior parietal gyrus (IPG)	L	− 40 − 42 42	1683	13.90
	Middle occipital gyrus (MOG)	L	− 26 − 76 34		11.95
	Middle frontal gyrus (MFG)	L	− 26 0 56	1266	13.09
	Supplementary motor area (SMA)	L	− 4 10 52		12.58
	Supplementary motor area (SMA)	R	8 8 62		9.55
	Inferior frontal gyrus-triangular part (IFGtriang)	L	− 40 28 24	1031	11.92
	Inferior frontal gyrus-opercular part (IFGoperc)	L	− 44 12 18		9.66
	Cerebellum_Crus1 (CERCRU1)	L	− 8 − 76 − 22	49	10.91
	Insula (INS)	L	− 34 18 2	221	10.65
	Middle frontal gyrus (MFG)	R	26 12 50	454	9.82
	Superior frontal gyrus-dorsolateral (SFG)	R	24 12 60		9.22
	Inferior frontal gyrus-triangular part (IFGtriang)	R	46 30 28	655	8.79
	Middle frontal gyrus (MFG)	R	44 44 28		8.12
	Cerebellum_6 (CER6)	R	30 − 62 − 30	291	8.37
	Middle frontal gyrus (MFG)	L	− 34 50 6	233	8.13
	Inferior frontal gyrus-opercular part (IFGoperc)	R	44 6 24	184	8.12
	Cerebellum_8 (CER8)	R	30 − 70 − 52	43	7.18
	Cerebellum_7b (CER7b)	R	22 − 74 − 46		5.43
	Pallidum_L	L	− 16 2 6	71	6.73

*MNI* Montreal Neurological Institute, *L* left, *R* right.

Significant brain activations were reported at the cluster level.

**Figure 4:**
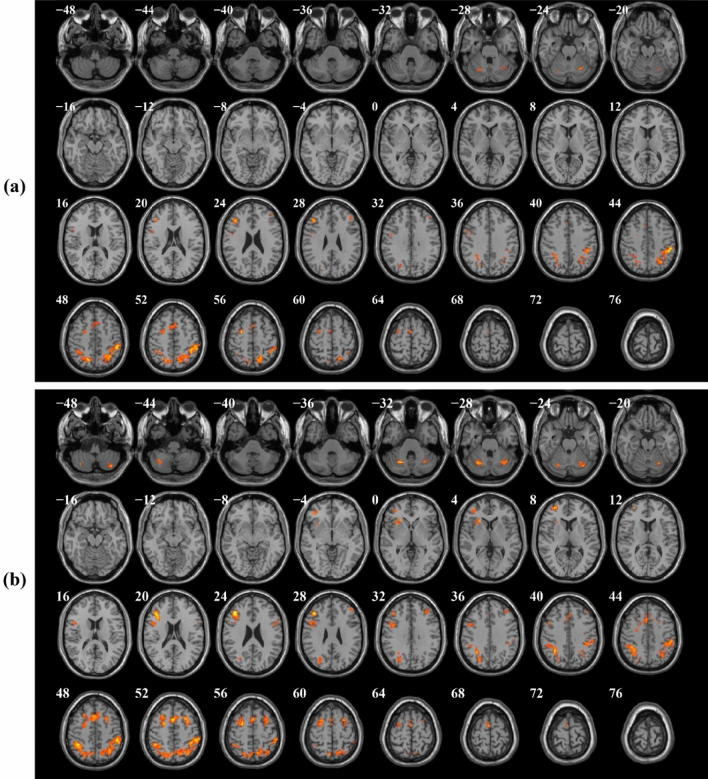
Significant activation patterns during each contrast of 2-back vs. 1-back, and 3-back vs. 1-back in healthy controls (one-sample t-tests, *p* < 0.001, uncorrected).

There were 9 and 13 activation clusters found for 2-back and 3-back vs. 1-back contrasts, respectively. So, the extended activation was also observed as the cognitive task difficulty increased. During 2-back, 3-back vs. 1-back contrasts, common regions of activation clusters were seen in the bilateral inferior parietal gyri, middle frontal gyri, left triangular part of inferior frontal gyri, left precentral, cerebellum_6, and supplementary motor area. For contrasts of 2-back vs. 1-back, and 3-back vs. 1-back, respectively, 2 (1585 voxels total), 1 (2610 voxels total) parietal clusters, 3 (294 voxels total), 4 (843 voxels total) frontal clusters, and 2 (87 voxels total), 4 (383 voxels total) cerebellum clusters were significantly activated. The activation in 3-back vs. 1-back was greater than in 2-back vs. 1-back and involved more regions of the left insula, cerebellum, and frontal lobe, including the left opercular and orbital parts of the inferior frontal gyrus, as well as the right superior frontal gyrus. Figure 4 and Table 3 show the patterns of activations during each contrast of 2-back and 3-back vs. 1-back separately.

**Table 3:** Clusters of brain regions that were significantly activated during 2-back vs. 1-back, and 3-back vs. 1-back contrasts with increasing task difficulty in a within group analysis (one-sample t-tests, *p* < 0.001, uncorrected).

Image contrast	Brain region	Side	Peak MNI coordinates (*x y z*)	Cluster extent	*T*-value
2-back vs. 1-back (activation clusters)	Superior parietal gyrus (SPG)	L	− 18 − 64 48	463	13.71
	Inferior parietal gyrus (IPG)	L	− 42 − 46 50		6.66
	Inferior parietal gyrus (IPG)	R	48 − 40 46	1122	11.95
	SupraMarginal gyrus (SMG)	R	50 − 32 44		9.68
	Middle frontal gyrus (MFG)	L	− 28 − 2 56	140	10.16
	Inferior frontal gyrus-triangular part (IFGtriang)	L	− 42 32 26	114	9.11
	Precentral gyrus (PreCG)	L	− 48 0 30	73	6.86
	Cerebellum_6 (CER6)	R	24 − 62 − 22	56	6.85
	Supplementary motor area (SMA)	L	− 2 − 2 64	181	6.35
	Supplementary motor area (SMA)	R	2 12 54		5.53
	Middle frontal gyrus (MFG)	R	42 38 30	40	6.09
	Cerebellum_6 (CER6)	L	− 26 − 64 − 30	31	5.69
3-back vs. 1-back (activation clusters)	Inferior frontal gyrus-triangular part (IFGtriang)	L	− 42 34 26	296	13.49
	Inferior parietal gyrus (IPG)	R	48 − 36 50	2610	13.22
	Inferior parietal gyrus (IPG)	L	− 42 − 44 50		11.10
	Cerebellum_Crus1 (CERCRU1)	L	− 32 − 64 − 30	139	11.94
	Cerebellum_6 (CER6)	L	− 20 − 62 − 32		6.57
	Middle frontal gyrus (MFG)	L	− 34 52 10	157	9.04
	IFG pars orbitalis (IFGorb)	L	− 44 40 − 4		7.02
	Supplementary motor area (SMA)	L	− 4 10 52	908	8.92
	Middle frontal gyrus (MFG)	L	− 26 4 54		8.58
	Cerebellum_7b (CER7b)	R	36 − 70 − 48	35	7.88
	Insula (INS)	L	− 26 24 2	107	7.73
	Middle frontal gyrus (MFG)	R	32 6 54	302	7.49
	Superior frontal gyrus-dorsolateral (SFG)	R	26 10 60		7.42
	Middle frontal gyrus (MFG)	R	44 44 26	88	6.96
	Cerebellum_6 (CER6)	R	24 − 64 − 22	164	6.91
	Precentral gyrus (PreCG)	L	− 48 10 38	277	6.49
	Inferior frontal gyrus-opercular part (IFGoperc)	L	− 50 10 18		6.36
	Supplementary motor area (SMA)	L	− 2 0 62	76	6.37
	Cerebellum_7b (CER7b)	L	− 34 − 64 − 46	45	6.19

*MNI* Montreal Neurological Institute, *L* left, *R* right.

Significant brain activations were reported at the cluster level.

**Figure 5:**
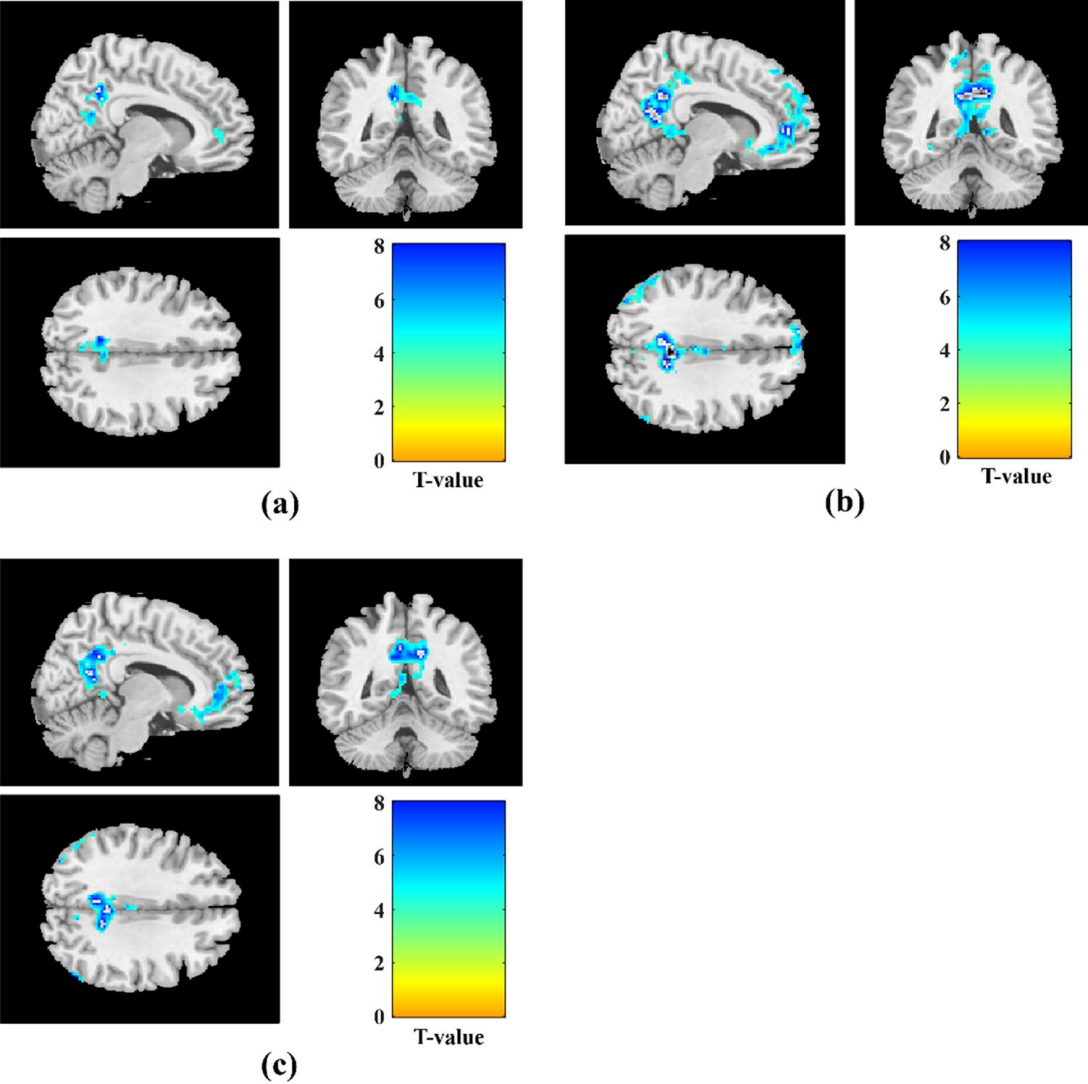
Significant clusters of deactivated brain regions during 1-back vs. 0-back, 2-back vs. 0-back, and 3-back vs. 0-back contrasts with increasing cognitive task load in healthy controls (one-sample t-tests, *p* < 0.001, uncorrected).

**Figure 6:**
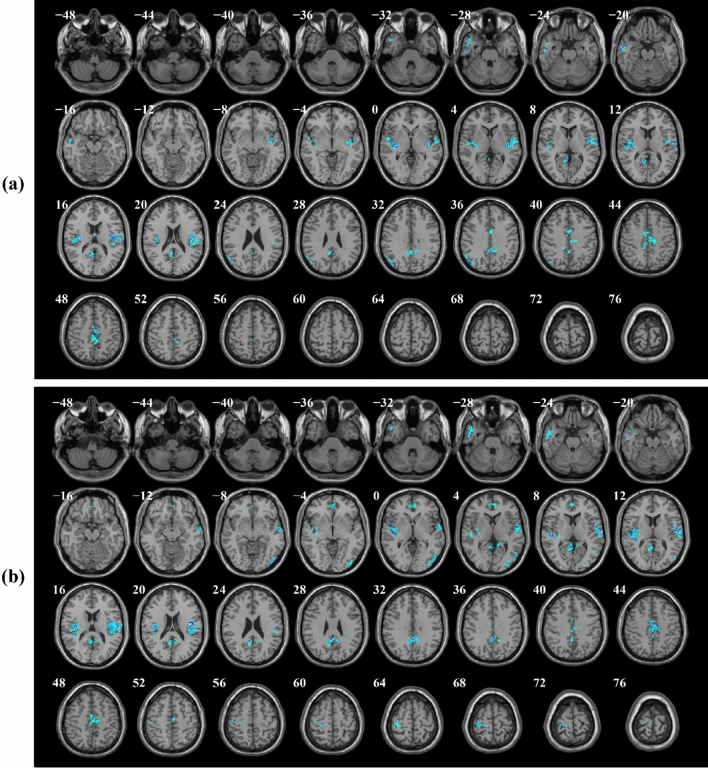
Significant deactivation patterns during each contrast of 2-back vs. 1-back, and 3-back vs. 1-back in healthy controls (one-sample t-tests, *p* < 0.001, uncorrected).

The patterns of deactivation during 1-back, 2-back, and 3-back vs. 0-back and 1-back contrasts also revealed significant clusters in the posterior cingulate gyrus (PCC), precuneus, angular, medial superior frontal gyrus, middle temporal gyrus (MTG), and superior temporal gyrus (STG), which are described as parts of the default mode network (DMN), as well as middle cingulate and paracingulate gyri (MCC), Heschl’s gyrus, fusiform, insula, and rolandic operculum areas. Clusters of brain areas that were significantly deactivated during the N-back task as cognitive load increased from 1-back to 3-back vs. 0-back conditions are shown in Fig. 5 and Table 4; similarly, Fig. 6 and Table 5 indicate brain area clusters that were significantly deactivated during the 2-back and 3-back vs. 1-back conditions.

**Table 4:** Clusters of brain regions that were significantly deactivated during 1-back vs. 0-back, 2-back vs. 0-back, and 3-back vs. 0-back contrasts with increasing task difficulty in a within group analysis (one-sample t-tests, *p* < 0.001, uncorrected).

Image contrast	Brain region	Side	Peak MNI coordinates (*x y z*)	Cluster extent	*T*-value
1-back vs. 0-back (deactivation clusters)	Middle cingulate and paracingulate (MCC)	L	− 14 − 48 36	285	10.67
	Posterior cingulate gyrus (PCC)	R	6 − 44 30		6.48
	Precuneus (PCUN)	L	− 6 − 62 32		5.85
	Angular gyrus (ANG)	L	− 42 − 68 34	113	8.82
	Pregenual anterior cingulate cortex (pACC)	L	− 6 46 6	161	8.62
	Pregenual anterior cingulate cortex (pACC)	R	2 48 10		7.76
	Superior frontal gyrus, medial (SFGmedial)	R	4 58 12		7.34
	Precuneus (PCUN)	R	4 − 54 14	358	8.49
	Precuneus (PCUN)	L	− 4 − 56 14		8.29
2-back vs. 0-back (deactivation clusters)	Middle cingulate and paracingulate (MCC)	L	0 − 44 34	3149	22.97
	Precuneus (PCUN)	L	− 6 − 50 14		14.40
	Posterior cingulate gyrus (PCC)	L	− 8 − 48 32		13.47
	Middle temporal gyrus (MTG)	L	− 52 2 − 26	550	13.40
	Heschl’s gyrus (HES)	R	50 − 14 4	440	12.55
	Superior temporal gyrus (STG)	R	58 − 6 0		8.00
	Insula (INS)	R	38 − 14 − 2		7.03
	Pregenual anterior cingulate cortex (pACC)	L	− 2 44 − 2	2634	11.46
	Superior frontal gyrus, medial orbital (PFCventmed)	R	10 48 − 8		11.08
	Insula (INS)	L	− 40 − 12 6	218	9.62
	Rolandic operculum (ROL)	L	− 42 − 16 22		6.53
	Fusiform gyrus (FFG)	L	− 24 − 34 − 18	334	9.41
	Parahippocampal gyrus (PHG)	L	− 32 − 42 − 10		9.23
	Hippocampus (HIP)	L	− 36 − 20 − 18		8.34
	Angular gyrus (ANG)	L	− 58 − 62 26	572	8.24
	Angular gyrus (ANG)	R	62 − 54 24	235	7.39
	Middle temporal gyrus (MTG)	R	50 − 72 20		6.87
	Cerebellum_Crus1 (CERCRU1)	R	30 − 76 − 34	218	7.15
	Cerebellum_Crus2 (CERCRU2)	R	24 − 88 − 32		6.78
3-back vs. 0-back (deactivation clusters)	Precuneus (PCUN)	L	− 8 − 50 16	1622	12.05
	Posterior cingulate gyrus (PCC)	L	− 2 − 40 32		10.61
	Middle cingulate and paracingulate (MCC)	R	10 − 46 34		10.04
	Superior frontal gyrus, medial (SFGmedial)	R	8 62 12	1540	10.49
	Pregenual anterior cingulate cortex (pACC)	L	− 2 46 0		9.64
	Superior frontal gyrus, medial orbital (PFCventmed)	R	6 26 − 14		9.58
	Middle cingulate and paracingulate (MCC)	R	14 − 20 44	240	10.18
	Supplementary motor area (SMA)	R	10 − 16 54		7.32
	Middle cingulate and paracingulate (MCC)	L	0 − 12 46		6.67
	Middle temporal gyrus (MTG)	L	− 52 2 − 26	513	9.97
	Temporal pole: middle temporal gyrus (TPOmid)	L	− 48 10 − 32		9.06
	Rolandic operculum (ROL)	L	− 40 − 16 16	215	9.04
	Superior temporal gyrus (STG)	L	− 58 − 24 10		6.40
	Angular gyrus (ANG)	R	52 − 68 34	287	8.00
	Middle temporal gyrus (MTG)	R	52 − 64 10		7.49
	Heschl’s gyrus (HES)	R	38 − 28 16	347	7.76
	Rolandic operculum (ROL)	R	62 − 16 14		7.17
	Superior temporal gyrus (STG)	R	54 − 24 8		6.60
	Temporal pole: superior temporal gyrus (TPOsup)	R	48 22 − 26	346	7.52
	Superior temporal gyrus (STG)	R	60 − 2 0		7.25
	Middle temporal gyrus (MTG)	R	50 4 − 28		7.15
	Angular gyrus (ANG)	L	− 48 − 70 38	260	7.23
	Middle occipital gyrus (MOG)	L	− 40 − 80 34		6.79

*MNI* Montreal Neurological Institute, *L* left, *R* right.

Significant brain deactivations were reported at the cluster level.

**Table 5:** Clusters of brain regions that were significantly deactivated during 2-back vs. 1-back, and 3-back vs. 1-back contrasts with increasing task difficulty in a within group analysis (one-sample t-tests, *p* < 0.001, uncorrected).

Image contrast	Brain region	Side	Peak MNI coordinates (*x y z*)	Cluster extent	*T*-value
2-back vs. 1-back (deactivation clusters)	Rolandic operculum (ROL)	R	40 − 12 18	805	14.86
	Superior temporal gyrus (STG)	R	62 − 14 8		8.54
	Insula (INS)	R	40 − 4 − 6		8.40
	Middle cingulate and paracingulate (MCC)	L	0 − 42 36	633	9.36
	Middle cingulate and paracingulate (MCC)	R	2 − 34 50		7.66
	Insula (INS)	L	− 36 − 18 0	503	9.34
	Rolandic operculum (ROL)	L	− 44 − 6 12		7.22
	Angular gyrus (ANG)	L	− 48 − 72 34	169	8.81
	Inferior parietal gyrus (IPG)	L	− 58 − 56 36		6.03
	Middle temporal gyrus (MTG)	L	− 52 − 4 − 22	189	7.76
	Temporal pole: middle temporal gyrus(TPOmid)	L	− 52 14 − 28		7.44
	Precuneus (PCUN)	L	− 8 − 54 14	159	7.37
3-back vs. 1-back (deactivation clusters)	Middle cingulate and paracingulate (MCC)	L	− 2 − 42 36	643	12.55
	Precuneus (PCUN)	R	6 − 46 18		9.96
	Superior temporal gyrus (STG)	R	62 − 14 10	897	11.98
	Rolandic operculum (ROL)	R	52 − 2 6		10.72
	Rolandic operculum (ROL)	L	− 40 − 14 18	614	10.81
	Superior temporal gyrus (STG)	L	− 50 − 10 − 4		9.69
	Insula (INS)	L	− 34 − 20 10		8.76
	Middle cingulate and paracingulate (MCC)	R	4 − 14 44	336	10.39
	Middle cingulate and paracingulate (MCC)	L	− 4 − 4 44		8.11
	Middle temporal gyrus (MTG)	R	48 − 68 2	270	9.09
	Inferior occipital gyrus (IOG)	R	46 − 78 − 8		8.77
	Middle occipital gyrus (MOG)	R	42 − 80 0		8.30
	Paracentral lobule (PCL)	L	− 16 − 30 72	215	8.67
	Postcentral gyrus (PoCG)	L	− 46 − 16 56		7.55
	Pregenual anterior cingulate cortex (pACC)	L	0 46 − 2	205	7.94
	Superior frontal gyrus, medial (SFGmedial)	R	4 50 4		5.46
	Temporal pole: middle temporal gyrus (TPOmid)	L	− 52 10 − 30	205	6.89
	Middle temporal gyrus (MTG)	L	− 60 − 14 − 20		6.64

*MNI* Montreal Neurological Institute, *L* left, *R* right.

Significant brain deactivations were reported at the cluster level.

Brain regions distributed in the frontoparietal lobes, cerebellum, insula, SMA and lenticular nucleus were included in the resulting patterns of activation map from the contrasts during the N-back task with different loads, which was consistent with previous research. The map of deactivation patterns of regions highlighted the majority of the DMN, which was also consistent with the findings of previous studies^3,15,18,20^.

These previously mentioned regions have been reported to play a role in diverse aspects of cognitive processes, such as the IPG (engagement in various mental functions, including maintenance of attention, visual presentation of objects, short-term and visuospatial working memory), MFG (involved in more complicated processes, working memory aspects, and attention control), the cerebellum (involvement in a wide range of memory, executive functions, and attention), and the insula (related to cognition, detection of salient changes in cognition, and effort to solve cognitive problems). The SMA and precentral gyrus (for the sensorimotor network) were also included in the N-back task due to their contribution to recording motor responses^3,15,18,20,25–33^.

An activation pattern was observed in the left superior frontal gyrus-dorsolateral (SFG) during the 3-back vs. 2-back contrast, but no clusters for deactivation patterns reached statistically significant differences. The superior frontal gyrus is thought to aid high-level cognitive functions, especially working memory^33^.

## Conclusion

This study aimed to identify brain recruitment patterns associated with cognitive load-dependent activation/deactivation during working memory task execution in healthy subjects. To achieve this goal, we used an fMRI protocol with a 3.0 T magnet and a Persian version of the N-back task with three levels of complexity. Within-group analysis under SPM12 revealed 7, 13, and 15 significant clusters of activation with increasing task difficulty in contrast-dependent brain activity maps during contrasts of 1-back, 2-back, and 3-back vs. 0-back. In line with the previous research findings, recruitment patterns were found in the frontoparietal lobes, cerebellum, insula, SMA, and lenticular nucleus, which were more related to regions involved in aspects of cognitive functions, and deactivation patterns were found in brain areas commonly described as being part of the DMN. This study could confirm the feasibility of using fMRI underlying N-back task performance with different levels of difficulty to assess the abnormal recruitment of regions in patients who suffer from cognitive impairments in brain disorders by comparing controls in future studies.

## Data Availability

The datasets used and analyzed during the current study are available from the corresponding author on reasonable request.
